# The Thermoelectric Performance of Monolayer Penta-NiN_2_ with Four-Phonon Scattering

**DOI:** 10.3390/ma19194162

**Published:** 2026-09-29

**Authors:** Hongmei Zheng, Yuqi Zeng, Honggang Zhang, Ping Wang, Chuanfu Li, Yangshun Lan

**Affiliations:** Key Laboratory of High Performance Scientific Computation, School of Science, Xihua University, Chengdu 610039, China

**Keywords:** two-dimensional materials, pentagonal, NiN_2_, four-phonon scattering, lattice thermal conductivity, thermoelectric performance

## Abstract

The exploration of two-dimensional semiconductors exhibiting poor lattice heat conduction offers a viable pathway for developing high-efficiency thermoelectric materials. Calculations incorporating four-phonon scattering reveal that monolayer penta-NiN_2_ possesses remarkably low lattice thermal conductivity, indicating its significant potential for thermoelectric applications. Here, we systematically evaluate the thermoelectric properties of monolayer penta-NiN_2_ by combining first-principles calculations with Boltzmann transport theory, with particular emphasis on the role of four-phonon scattering. Structural analysis confirms its stability and semiconducting nature with a direct band gap. The inclusion of four-phonon scattering leads to a pronounced reduction in the lattice thermal conductivity, from 51.87 W/mK (three-phonon only) to 4.27 W/mK at room temperature. Electronic transport analysis reveals that hole doping yields a substantially higher power factor (2659 μW/cm/K^2^) than electron doping (1141 μW/cm/K^2^). Consequently, the optimal *ZT* reaches 7.57 for hole doping and 1.83 for electron doping with four-phonon processes taken into account, both significantly higher than those obtained under the three-phonon-only scheme. These results establish monolayer penta-NiN_2_ as a promising thermoelectric candidate and highlight the critical importance of incorporating four-phonon scattering for a reliable description of thermal transport in penta-2D materials.

## 1. Introduction

A large amount of waste heat is generated in industrial processes, with temperatures mainly ranging from 400 to 900 K [1]. The recovery and utilization of such waste heat has long been a focal point in energy harvesting research. Environmentally friendly thermoelectric (TE) materials offer unique potential for waste heat recovery [2]. In a TE material, a temperature difference drives charge carriers and produces an electrical voltage through the Seebeck effect, thereby allowing heat to be directly converted into electricity. The efficiency of this energy conversion is evaluated using the figure of merit (*ZT* = *S*^2^*σT*/*κ*). *S* is the Seebeck coefficient, *σ* the electrical conductivity, *T* the absolute temperature, and *κ* the total thermal conductivity. The total thermal conductivity *κ* is given by *κ* = *κ*_L_ + *κ*_e,_ where *κ*_L_ and *κ*_e_ denote the lattice and electronic contributions, respectively. A high thermoelectric efficiency requires a large power factor *S*^2^*σ* and a low thermal conductivity *κ*. However, the inherent trade-off among *S*, *σ*, and *κ*_e_ poses a fundamental challenge in achieving high *ZT* values [3]. Therefore, balancing *S*, *σ*, and *κ*_e_ while reducing *κ*_L_ appears to be a viable strategy for attaining high *ZT* values. Computational materials design has become an effective approach for identifying promising thermoelectric compounds, while low-dimensional systems have also attracted considerable interest because of their tunable electronic and phonon transport properties [4,5,6].

Following the proposal of penta-graphene in 2015, pentagonal two-dimensional (penta-2D) materials have been explored because of their distinctive structural and physical properties. Penta-2D materials are constructed from Cairo pentagonal tiling or its variants [7], exhibiting intrinsically buckled or puckered geometries [8]. These materials exhibit unique mechanical and electronic properties [9,10,11], making them potential candidates for fields such as energy conversion and storage, electronic and optoelectronic devices, sensors, and spintronics. Specifically, unlike traditional 2D materials, penta-2D materials exhibit particular thermal transport properties, giving them considerable potential for efficient TE conversion. Penta-2D materials generally exhibit low lattice thermal conductivity because their buckled pentagonal geometry suppresses phonon harmonic interaction and strengthens anharmonic scattering [12,13,14,15,16]. Concurrently, these materials maintain favorable electrical transport properties, benefiting from multi-valley degeneracy near the band edges that enhances the Seebeck coefficient [16,17], and weak electron-phonon coupling that ensures high carrier mobility [18,19]. The inherent in-plane anisotropy further provides additional degrees of freedom for performance optimization through crystallographic orientation selection and strain engineering [20,21,22].

The integration of first-principles calculations with Boltzmann transport theory provides a well-established framework for evaluating thermoelectric properties [23]. This approach has yielded remarkably high *ZT* values in several penta-2D systems, owing to the advantages of these materials discussed above. The penta-Sb_2_Sn and penta-Sb_2_Ge monolayers achieve *ZT* values of 8.0 and 7.37 for *p*-type doping at 700 K respectively [15], while penta-Bi_2_Ge yields a *ZT* of 6.96 under the same conditions [16]. For penta-PdTe_2_, the room-temperature *ZT* values reach 6.6 in the *p*-type regime and 4.4 in the *n*-type regime [19]. Even the elemental penta-silicene demonstrates a *ZT* of approximately 3.4 at room temperature [24]. These remarkably high *ZT* values have stimulated ongoing efforts to discover additional high-performance thermoelectric candidates in penta-2D systems.

Penta-NiN_2_, a representative member of the penta-2D family, has shown promise in applications such as electrode materials [25], catechol sensing [26], and bifunctional oxygen electrocatalysis [27]. An early notable feature of penta-NiN_2_ was its high lattice thermal conductivity [28]. However, a recent study reveals that considering higher-order phonon scattering processes significantly affects its thermal transport properties. When four-phonon (4ph) scattering is taken into account, the room-temperature intrinsic lattice thermal conductivity drops to 11.67 W/mK, 89.32% lower than that predicted using only three-phonon (3ph) processes [29]. This finding suggests that penta-NiN_2_ may also possess high thermoelectric performance, demonstrating its potential in the field of thermoelectric materials. Nevertheless, the TE properties of penta-NiN_2_ remain largely unexplored.

In this work, the TE properties of monolayer penta-NiN_2_ are examined within a theoretical framework that integrates first-principles calculations with Boltzmann transport theory. The lattice thermal conductivity *κ*_L_ is determined by solving the phonon Boltzmann transport equation, with both 3ph and 4ph scattering processes incorporated. The electronic transport coefficients, characterized by Seebeck coefficient *S*, electrical conductivity *σ*, and electronic thermal conductivity *κ*_e_, are evaluated using semiclassical Boltzmann transport theory. On the basis of the calculated thermal and electronic transport characteristics, we further evaluate the thermoelectric performance of monolayer penta-NiN_2_ and identify the key physical factors responsible for its conversion efficiency.

## 2. Computational Methods

Density functional theory (DFT) calculations are conducted using the Vienna ab initio simulation package (VASP 5.4.4) with the version of 5.4.4 [30,31,32]. The exchange-correlation functional is described by the generalized gradient approximation (GGA) with the Perdew–Burke–Ernzerhof (PBE) formulation [33]. Structural optimization is performed with a plane-wave cutoff energy of 800 eV and a 12 × 12 × 1 Monkhorst–Pack *k*-point mesh for Brillouin-zone sampling [34]. To minimize interactions between periodically repeated layers, a vacuum spacing exceeding 15 Å is introduced along the *c*-direction. During optimization, the out-of-plane lattice parameter *c* is kept fixed, whereas the in-plane lattice parameters *a* and *b*, together with all atomic coordinates, are fully relaxed until the residual Hellmann-Feynman forces on each atom are below 10^−4^ eV/Å.

The electronic band structure is evaluated using both the PBE functional and the HSE06 hybrid functional [35]. Phonon dispersions and second-order interatomic force constants (IFCs) are determined through the finite displacement approach with a 4 × 4 × 1 supercell [36]. The third- and fourth-order IFCs are evaluated using 4 × 4 × 1 and 3 × 3 × 1 supercells, respectively, with anharmonic interactions included up to the third-nearest neighbor shell. The lattice thermal conductivity *κ*_L_ is evaluated through an iterative solution of the phonon Boltzmann transport equation (BTE) using the ShengBTE with the version of 1.5.1 and FourPhonon with the version of 1.0 packages [37,38]. In the BTE calculations, 75 × 75 × 1 and 16 × 16 × 1 *q*-point meshes are used for the 3ph-only and coupled 3ph and 4ph calculations, respectively. Electronic transport properties, represented by *S*, *σ* and *κ*_e_, are evaluated within semiclassical Boltzmann transport theory using the BoltzTraP code with the version of 1.2.5 [39], based on the PBE electronic band structure. For the calculation of *κ*_L_, *σ* and *κ*_e_, an effective thickness of 2.93 Å is consistently adopted, based on the interlayer spacing of bilayer penta-NiN_2_ obtained including van der Waals corrections [40]. The lattice thermal conductivity is calculated over the temperature range of 100–1200 K. The electronic transport properties and thermoelectric figure of merit *ZT* are evaluated at 300 K. The thermoelectric figure of merit *ZT* is subsequently evaluated as *ZT* = *S*^2^*σT*/(*κ*_L_+*κ*_e_).

## 3. Results and Discussion

**Figure 1:**
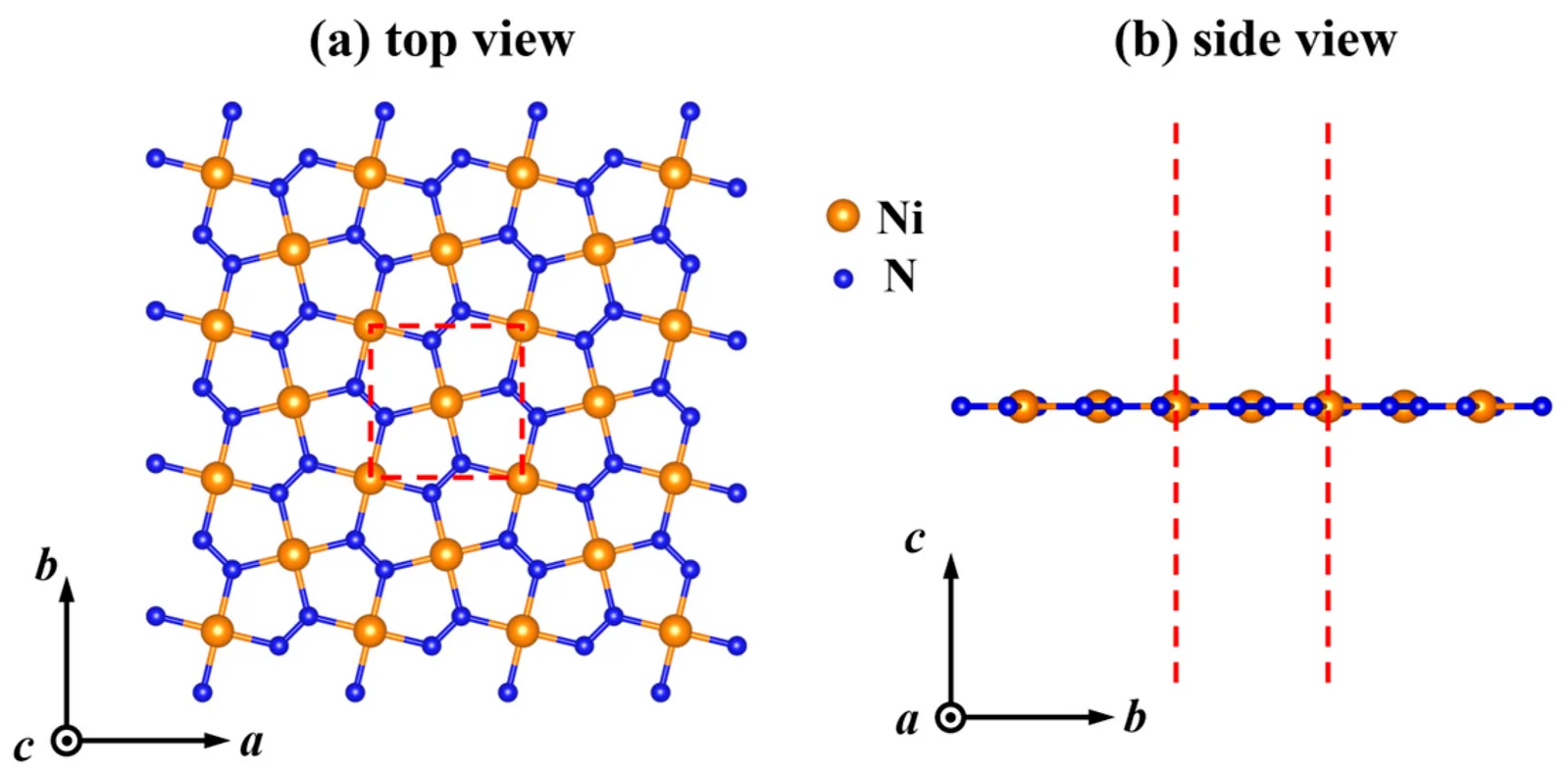
Relaxed structure of monolayer penta-NiN_2_ viewed from (**a**) the top and (**b**) the side view. The primitive cell is outlined by red dashed lines.

The monolayer penta-NiN_2_ crystallizes in a tetragonal lattice with the space group *P*4/*mbm* (No. 127), and its primitive cell consists of two Ni and four N atoms. The optimized geometry of monolayer penta-NiN_2_ is presented in Figure 1. As illustrated in the top view (Figure 1a), each pentagonal unit consists of three N atoms and two Ni atoms. The side view (Figure 1b) confirms that all atoms remain coplanar. After structural relaxation, the in-plane lattice parameters are *a* = *b* = 4.53 Å, while the Ni-N bond length and the N-N dimer distance are 1.88 Å and 1.24 Å, respectively. These structural parameters agree well with previously reported theoretical values.

**Figure 2:**
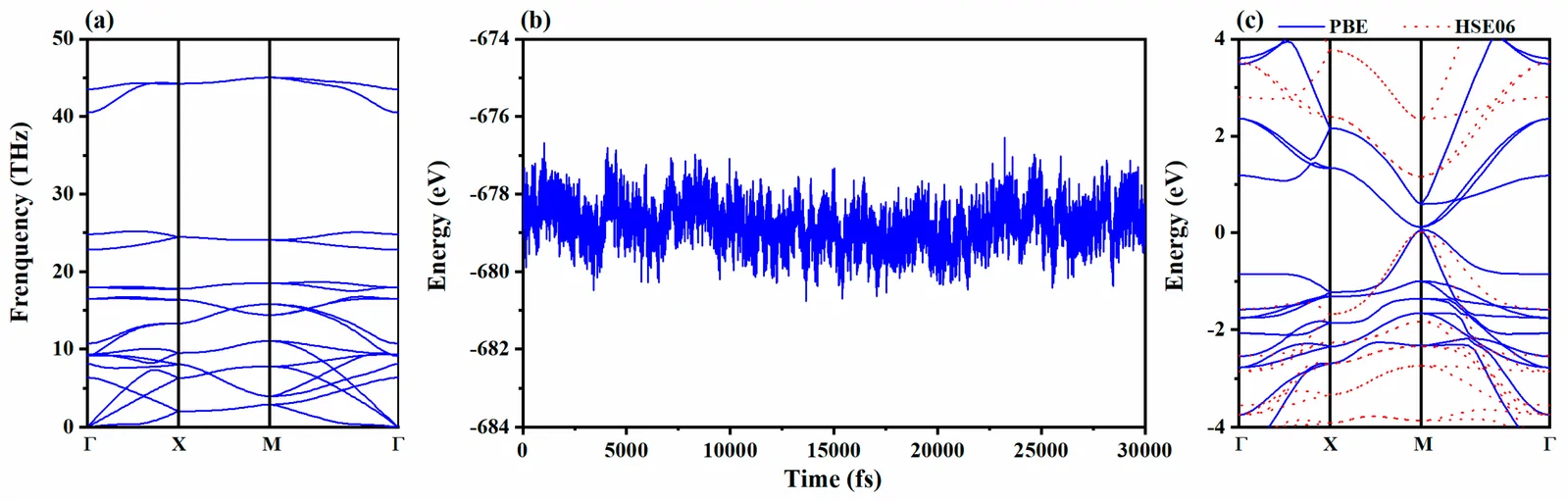
Calculated (**a**) phonon spectrum, (**b**) total energy fluctuation during the AIMD simulation at 300 K, and (**c**) electronic band structure of monolayer penta-NiN_2_. In (**c**), the blue solid lines and red dashed lines denote the band structures obtained using the PBE and HSE06 functionals, respectively.

Before investigating the thermoelectric performance of the monolayer penta-NiN_2_, its structural stability and semiconducting properties should first be established. The dynamical stability is first examined through its phonon spectrum, as shown in Figure 2a. No imaginary phonon modes are observed along the high-symmetry paths of the Brillouin zone, demonstrating its dynamical stability. The thermal stability is further assessed by ab initio molecular dynamics (AIMD) simulations using a 4 × 4 × 1 supercell at 300 K for 30 ps. As presented in Figure 2b, the total energy exhibits only small fluctuations throughout the simulation. Meanwhile, the atomic configuration remains intact without noticeable structural reconstruction or bond breaking, indicating good thermal stability at room temperature. Finally, the mechanical stability is examined through the calculated elastic constants listed in Table 1. The obtained elastic constants satisfy the Born stability criteria for a two-dimensional lattice (*C*_11_ > 0, *C*_66_ > 0, and *C*_11_ − *C*_12_ > 0), confirming the mechanical stability. The electronic band structures obtained using the PBE and HSE06 functionals are presented in Figure 2c. The PBE result, represented by the blue solid lines, yields a narrow band gap of 0.05 eV. When the HSE06 hybrid functional is employed, the band gap increases markedly to 1.14 eV, as indicated by the red dashed lines. Both calculations identify monolayer penta-NiN_2_ as a direct-gap semiconductor, with the valence band maximum (VBM) and conduction band minimum (CBM) both located at the high-symmetry M point.

**Figure 3:**
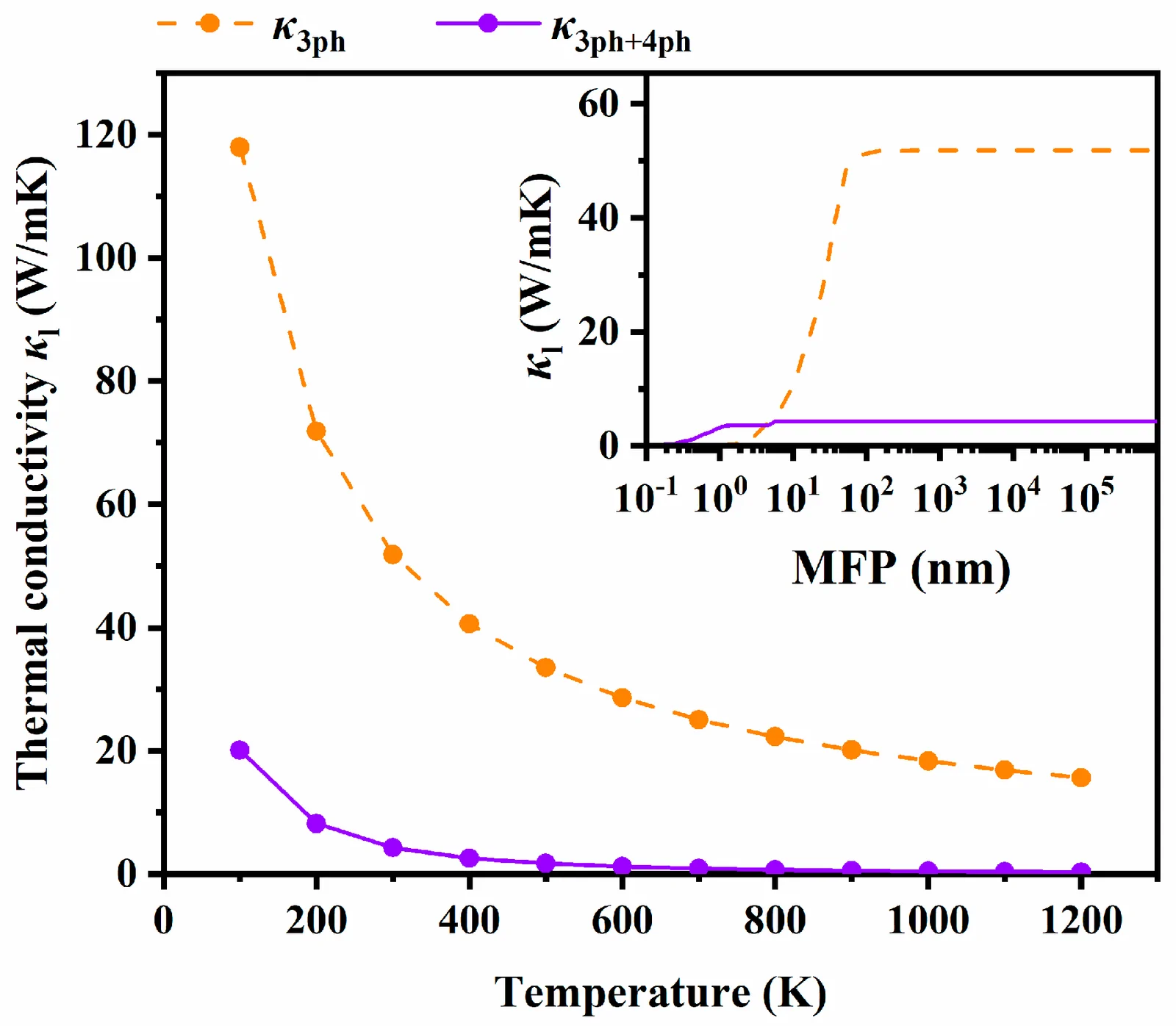
Lattice thermal conductivity *κ*_L_ of monolayer penta-NiN_2_ as a function of temperature, considering 3ph scattering alone and combined 3ph and 4ph scattering. The inset shows the cumulative *κ*_L_ as a function of phonon MFP at 300 K under the two scattering conditions.

As discussed in the Introduction, low lattice thermal conductivity is a key characteristic in the search for thermoelectric materials with high *ZT* values. Accordingly, the lattice thermal conductivity of monolayer penta-NiN_2_ is further investigated. Figure 3 shows the temperature dependence of *κ*_L_ obtained with 3ph scattering alone and with both 3ph and 4ph processes included. In both cases, *κ*_L_ decreases with increasing temperature, consistent with the typical behavior dominated by phonon scattering. The inclusion of 4ph processes leads to a pronounced suppression of *κ*_L_. For instance, at 300 K, *κ*_L_ decreases from 51.87 W/mK in the 3ph-only calculation to 4.27 W/mK when 4ph scattering is taken into account, corresponding to a reduction of approximately 92%. Although the resulting *κ*_L_ is lower than the previously reported value of 11.67 W/mK [29], both studies consistently demonstrate the strong suppression of lattice thermal transport induced by 4ph scattering. The 4ph scattering also markedly shortens the characteristic phonon mean free path (MFP), as illustrated in the inset of Figure 3. The MFP corresponding to 50% of the cumulative thermal conductivity decreases from over 20 nm (3ph-only) to less than 0.5 nm (including both 3ph and 4ph scattering), indicating that the thermal transport becomes highly sensitive to geometrical confinement.

**Figure 4:**
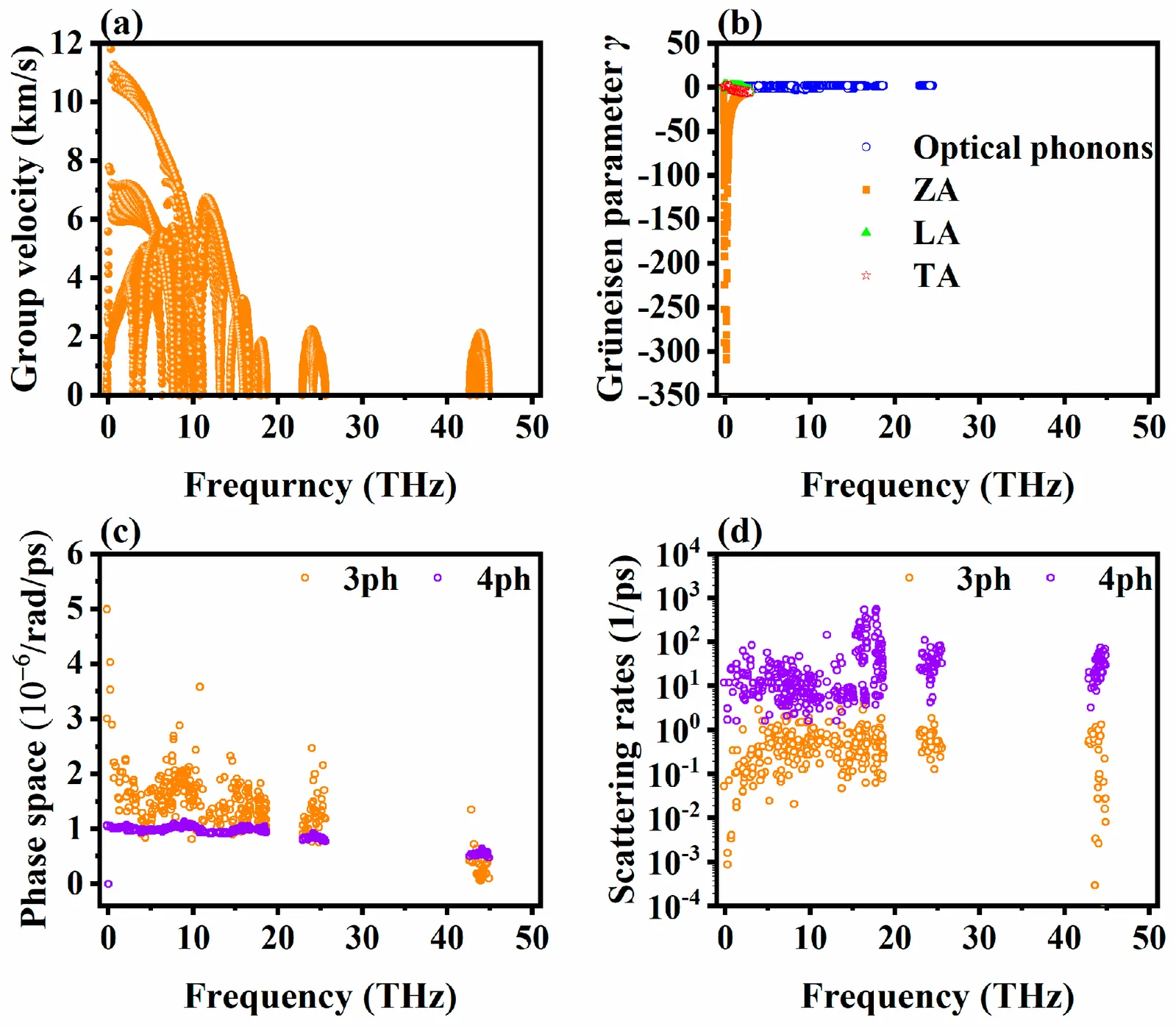
Frequency-dependent (**a**) phonon group velocity, (**b**) Grüneisen parameter *γ*, (**c**) scattering phase space and (**d**) scattering rates of monolayer penta-NiN_2_ at 300 K. The 3ph and 4ph contributions are compared in (**c**,**d**). In (**b**), TA, LA and ZA denote the transverse acoustic, longitudinal acoustic, and out-of-plane acoustic phonon modes, respectively.

To clarify the microscopic origin of the low *κ*_L_ in monolayer penta-NiN_2_, the frequency-dependent phonon group velocity, Grüneisen parameter γ, scattering phase space, and scattering rates are examined at 300 K, as presented in Figure 4. Among these quantities, the phonon group velocity and Grüneisen parameter γ are intrinsic properties and independent of the scattering order. As presented in Figure 4a, the acoustic phonon branches exhibit remarkably high group velocities, particularly in the low-frequency region, indicating efficient phonon propagation that would typically enhance lattice thermal conductivity. However, this effect is strongly counteracted by pronounced lattice anharmonicity. The mode-resolved Grüneisen parameter γ in Figure 4b shows particularly large absolute values for the low-frequency out-of-plane acoustic (ZA) modes, indicating strong anharmonic interactions and consequently enhanced phonon-phonon scattering. This strong anharmonicity effectively offsets the benefit of high group velocities, ultimately suppressing *κ*_L_ and yielding the observed low lattice thermal conductivity. To further explore the microscopic differences between 3ph and 4ph processes, their frequency-dependent scattering phase spaces and scattering rates are presented in Figure 4c and Figure 4d, respectively. The scattering phase space reflects the number of available channels that satisfy energy and momentum conservation. As presented in Figure 4c, the 4ph phase space (P4) is generally smaller than the corresponding 3ph phase space (P3), particularly in the low- and intermediate-frequency regions, suggesting that 3ph scattering is statistically more probable. Nevertheless, P4 remains sufficiently large to support appreciable phonon interactions, indicating that 4ph channels cannot be neglected. The 4ph scattering rates shown in Figure 4d exceed the 3ph rates over nearly the entire frequency range. Despite the smaller phase space, the much larger 4ph scattering rates indicate strong higher-order anharmonic interactions. Consequently, 4ph scattering plays a major role in shortening phonon lifetimes and strongly suppressing the lattice thermal conductivity of monolayer penta-NiN_2_.

**Figure 5:**
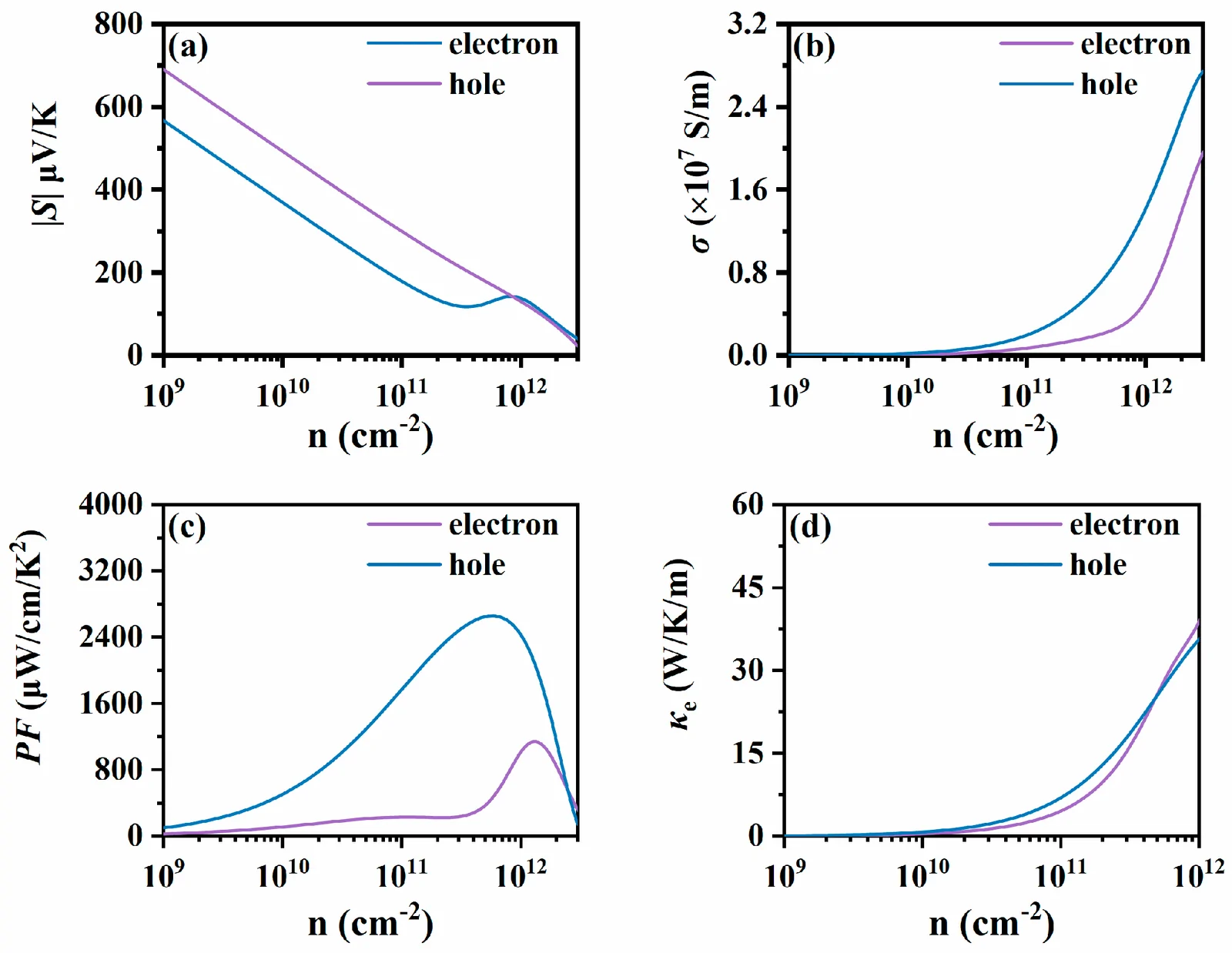
The calculated (**a**) Seebeck coefficient |*S*|, (**b**) electrical conductivity *σ*, (**c**) power factor *PF*, and (**d**) electronic thermal conductivity *κ*_e_ of monolayer penta-NiN_2_ as functions of carrier concentration for both electron and hole doping at 300 K.

Subsequently, the electronic transport coefficients of monolayer penta-NiN_2_ as a function of carrier concentration at 300 K are presented in Figure 5 for both electron and hole doping. A constant carrier relaxation time of *τ* = 10 fs is adopted at room temperature for the evaluation of the electrical conductivity *σ*, power factor *PF* = *S*^2^*σ*, and electronic thermal conductivity *κ*_e_ [14]. As shown in Figure 5a, the magnitude of |*S*| generally decreases with increasing carrier concentration for both doping types, consistent with the characteristic behavior of degenerate semiconductors [15]. Over most of the concentration range, hole doping gives larger |*S*| values than electron doping. In contrast, as shown in Figure 5b, *σ* increases monotonically with carrier concentration. Under the same relaxation time approximation, the higher electrical conductivity observed for hole doping indicates more favorable electronic transport than that for electron doping. The resulting power factor is shown in Figure 5c. Owing to the competing variations of |*S*| and *σ*, the *PF* reaches a maximum at an optimal carrier concentration rather than varying monotonically for both doping types. The optimal carrier concentrations are 6.0 × 10^11^ cm^−2^ for hole doping and 1.3 × 10^12^ cm^−2^ for electron doping, yielding optimal *PF* values of 2659 μW/cm/K^2^ and 1141 μW/cm/K^2^, respectively. The substantially higher *PF* for hole doping indicates that *p*-type doping is more favorable for achieving a high power factor. As shown in Figure 5d, the electronic thermal conductivity *κ*_e_ also increases monotonically with carrier concentration for both doping types. At low carrier concentrations, hole doping gives a higher *κ*_e_ than electron doping, while the opposite trend is observed at high carrier concentrations. Nevertheless, the electronic thermal conductivity does not differ significantly between the two doping types over the entire carrier concentration range studied. Therefore, the substantially larger *PF* together with the comparable *κ*_e_ suggests that hole doping is more favorable for achieving high thermoelectric performance in monolayer penta-NiN_2_.

**Figure 6:**
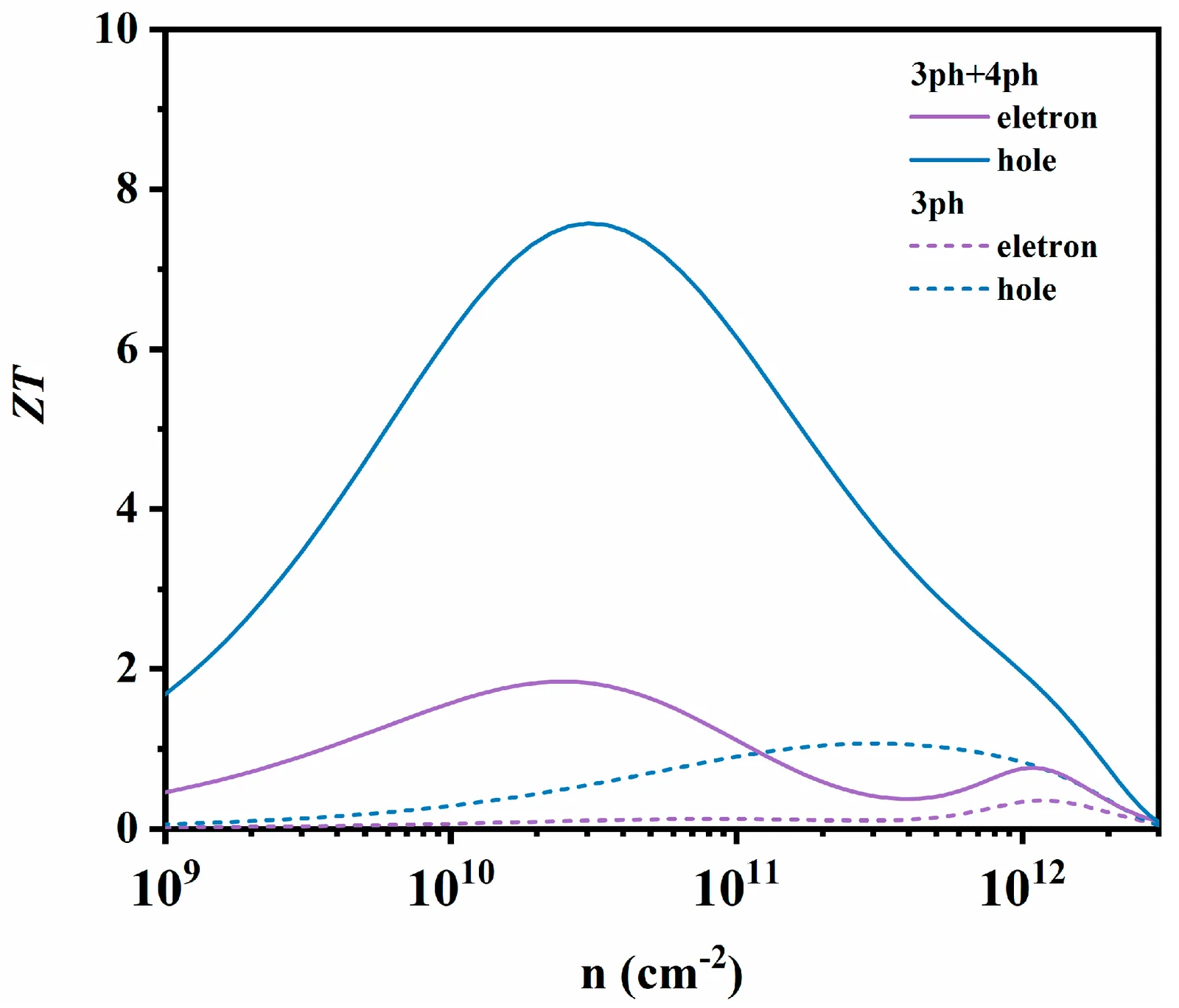
Thermoelectric figure of merit *ZT* of monolayer penta-NiN_2_ as a function of carrier concentration for electron and hole doping at 300 K, considering 3ph scattering alone and combined 3ph and 4ph scattering.

Finally, the thermoelectric figure of merit *ZT* as a function of carrier concentration at 300 K is presented in Figure 6 for both electron and hole doping using the lattice thermal conductivity obtained from the 3ph-only and combined 3ph and 4ph calculations. Under both scattering treatments, hole doping yields higher *ZT* values than electron doping, consistent with the superior electronic transport performance discussed above. For both doping types, the optimal *ZT* values obtained by including both 3ph and 4ph scattering are significantly higher than those obtained by considering only 3ph scattering, highlighting the substantial suppression of thermal transport induced by 4ph scattering. Specifically, the maximum *ZT* values and the corresponding carrier concentrations for hole and electron doping under the 3ph scattering alone and combined 3ph and 4ph scattering are summarized in Table 2. As shown in Table 2, at the adopted relaxation time of 10 fs, considering only 3ph scattering, hole doping achieves a *ZT* of 1.07 at an optimal carrier concentration of approximately 3.1 × 10^11^ cm^−2^, while electron doping yields a *ZT* of 0.35 at about 1.2 × 10^12^ cm^−2^. When both 3ph and 4ph scattering are included, hole doping reaches a *ZT* of 7.57 at approximately 3.0 × 10^10^ cm^−2^, whereas electron doping achieves a *ZT* of 1.83 at about 2.4 × 10^10^ cm^−2^. To assess the sensitivity of the predicted thermoelectric performance to the assumed carrier relaxation time *τ*, additional *ZT* calculations are performed using *τ* = 5, 15, and 20 fs, in addition to the adopted value of 10 fs, and the corresponding results are also summarized in Table 2. Although the absolute *ZT* values increase with increasing *τ*, the main qualitative trends remain unchanged over the examined range: hole doping consistently yields higher *ZT* values than electron doping, and the inclusion of 4ph scattering consistently results in substantially higher *ZT* values than those obtained with 3ph scattering alone. These results indicate that, although the absolute magnitude of *ZT* depends on the assumed relaxation time, the relative thermoelectric trends are robust against reasonable variations in *τ*. These results demonstrate the pronounced advantage of the *p*-type regime for thermoelectric performance in monolayer penta-NiN_2_. Moreover, when 4ph scattering is taken into account, not only is the *ZT* value significantly enhanced, but the optimal carrier concentration is also substantially reduced, which is more favorable for practical applications. Nevertheless, because the electronic transport calculations are based on the PBE band structure, which substantially underestimates the band gap compared with HSE06, the absolute *ZT* values may be subject to uncertainty associated with the possible overestimation of bipolar transport. Therefore, while the calculated *ZT* values provide useful quantitative estimates, greater emphasis should be placed on the overall transport trends and relative thermoelectric performance.

## 4. Conclusions

In summary, the thermoelectric properties of monolayer penta-NiN_2_ are systematically evaluated through first-principles calculations and Boltzmann transport theory, with particular attention to 4ph scattering. The calculated phonon dispersion, AIMD simulations, and elastic constants confirm its dynamical, thermal, and mechanical stability. Electronic structure calculations further identify it as a direct band gap semiconductor with both the VBM and CBM located at the high-symmetry M point. The 4ph scattering is found to strongly suppress lattice thermal transport. At room temperature, the lattice thermal conductivity decreases from 51.87 W/mK under 3ph scattering alone to 4.27 W/mK when both 3ph and 4ph processes are included, corresponding to a reduction of approximately 92%. The extremely low thermal conductivity originates from pronounced lattice anharmonicity, particularly that associated with low-frequency ZA phonons, together with strong 4ph scattering rates over a broad frequency range. Electronic transport analysis reveals that hole doping yields a substantially higher power factor (2659 μW/cm/K^2^) compared to electron doping (1141 μW/cm/K^2^). Accordingly, monolayer penta-NiN_2_ exhibits remarkably high thermoelectric performance under hole doping. When 4ph scattering is included, the optimal *ZT* reaches 7.57 for hole doping and 1.83 for electron doping, both significantly higher than those obtained under the 3ph-only scheme. These findings demonstrate the strong thermoelectric potential of monolayer penta-NiN_2_ and emphasize the necessity of higher-order phonon scattering for a reliable description of thermal transport in penta-2D materials.

## Figures and Tables

**Table 1:** Elastic constants of monolayer penta-NiN_2_ (N/m).

*C*_11_	*C*_12_	*C*_66_
174.18	22.81	44.47

**Table 2:** Maximum *ZT* values, *ZT*_max_, and corresponding optimal carrier concentrations, *n*_opt_ (cm^−2^), for hole and electron doping at 300 K under different carrier relaxation times *τ*, considering 3ph scattering alone and combined 3ph and 4ph (3ph + 4ph) scattering. Values in parentheses denote *n*_opt_.

Scattering Processes	Doping Type	*ZT*_max_ (*n*_opt_) with *τ* = 5 fs	*ZT*_max_ (*n*_opt_) with *τ* = 10 fs	*ZT*_max_ (*n*_opt_) with *τ* = 15 fs	*ZT*_max_ (*n*_opt_) with *τ* = 20 fs
3ph	Hole	0.62 (4.1× 10^11^)	1.07 (3.1× 10^11^)	1.43 (2.4× 10^11^)	1.74 (2.1× 10^11^)
3ph	Electron	0.23 (1.2 × 10^12^)	0.35 (1.2 × 10^12^)	0.43 (1.2 × 10^12^)	0.49 (1.2 × 10^12^)
3ph + 4ph	Hole	5.52 (4.8 × 10^10^)	7.57 (3.0 × 10^10^)	8.98 (2.2 × 10^10^)	10.06 (1.9 × 10^10^)
3ph + 4ph	Electron	1.17 (3.6 × 10^10^)	1.83 (2.4 × 10^10^)	2.35 (2.0 × 10^10^)	2.76 (1.8 × 10^10^)

## Data Availability

The original contributions presented in this study are included in the article. Further inquiries can be directed to the corresponding author.
